# Youssef's syndrome: Is there a better way to diagnose?

**DOI:** 10.4103/0970-1591.40631

**Published:** 2008

**Authors:** R. Shanmugasundaram, Ganesh Gopalakrishnan, Nitin S. Kekre

**Affiliations:** Department of Urology, Christian Medical College, Vellore, Tamil Nadu, India

**Keywords:** Menouria, magnetic resonance imaging, vesicouterine fistula

## Abstract

Vesicouterine fistula (VUF) the least common of the urogynecological fistulae. Hysterosalphingography is the gold standard investigation in demonstrating the fistulous track. It is an invasive investigation. We report a case of VUF where magnetic resonance imaging was useful to diagnose the condition in a non-invasive manner especially when the clinical picture was confusing.

## INTRODUCTION

Youssef's syndrome consists of cyclic hematuria, absence of vaginal bleeding (amenorrhoea), menouria, and urinary continence due to vesicouterine fistula (VUF). Youssef's syndrome has varied clinical presentation. Hysterography and cystography are commonly used to diagnose this condition. In this modern era, noninvasive investigations like computed tomography (CT) and magnetic resonance imaging (MRI) are being used for diagnosis. They are also useful to establish the diagnosis when clinical presentation is vague as it was in our case. This article emphasizes the role of MRI in the diagnosis of Youssef's syndrome.

## CASE REPORT

**Figure 1:**
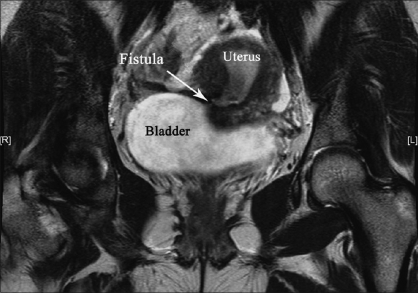
Coronal T2-weighted HR/SENSE MR image depicting the abnormal hypointense out pouching and fistulous tract (*arrow*) from posterior wall of bladder to anterolateral wall of uterus above the isthmus. Endometrial cavity is being opacified (hyperintense) with urine

A 27-year-old multiparous lady presented with cyclic hematuria of 2 years duration following lower segment cesarean section (LSCS). She also complained of intermittent wetness of vagina. Physical examination was unremarkable except for a well-healed Pfannensteil scar. There was no urine leak from the anterior vaginal wall or cervix during the speculum examination. In view of the past history of LSCS and menouria, diagnosis of VUF was considered. The MRI delineated the communication between the bladder and uterus above the isthmus [Figure 1] without any need for other invasive investigations. Cystoscopy showed a 5 × 5 mm erythematous area in the posterior wall of bladder 5 cm above the interureteric ridge. Methylene blue test was negative. Biopsy of the erythematous area revealed bladder mucosa with no significant lesion. She has been advised surgical exploration and disconnection of the fistula.

## DISCUSSION

**Figure 2:**
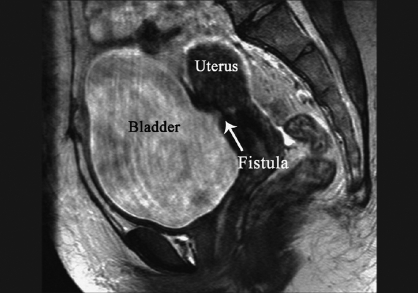
Sagittal T2-weighted HR/SENSE MR image (TR/TE, 4774/90) shows abnormal hypointense area (*arrow*) in the posterior wall of bladder communicating with uterus above the isthmus

In 1957, Youssef described a syndrome comprising of cyclic hematuria, amenorrhoea, menouria, and complete urinary continence in a patient who had LSCS.[1] The VUFs are among the least common urogynaecological fistulas. The VUF also occurs following high vaginal forceps-aided delivery, external cephalic version, curettage or manual removal of the placenta, placenta percreta, myomectomy, uterine rupture due to obstructed labor, uterine artery embolization, perforation of an intrauterine device, and brachytherapy for carcinoma of cervix. The LSCS is the single most common cause of VUF.[2] Amenorrhea, cyclic hematuria without urinary incontinence in combination with a history of LSCS has been described as pathognomonic of VUF.[3] The clinical presentation is often nonspecific and findings on examination classically used to depict the fistula may be negative, leading to considerable delay in diagnosis.[4] The VUF may not manifest with constant urinary incontinence because of a functional sphincter at the internal uterine os. Urinary incontinence occurs if the level of the VUF is at or below the internal os or if the os is incompetent.[3] In our case scanty urine leak occurred even in the presence of competent os with fistula communicating with uterus above isthmus. The diagnosis of VUF is often confirmed by imaging studies and cystoscopy. Cystoscopy even when repeated, can fail to confirm the fistula.[1] Methylene blue instilled into the uterine cavity or through the urethra or through catheterization of a visible lesion in the bladder wall can confirm the fistula. This test, however, does not show directly the fistulous tract and its specific location. Moreover, this test can be negative in patients with a long and tortuous tract.[1] Radiographically, both cystography and hysterography have been used in the diagnosis of VUFs. In Tancer's review of published reports, he found that hysterography was the most reliable diagnostic technique.[3] Intravenous urography can show the fistula when contrast medium enters the vagina, but distinguishing vesicovaginal and vesicouterine fistulae is difficult. Although VUFs are difficult to diagnose sonographically, Park *et al*. reported that sonography can demonstrate the fistulous tract as double echogenic lines between the endometrium of the anterior wall of the uterine body and the mucosal layer of the posterior wall of the bladder.[2] However, sonography has inherent difficulty in differentiating the VUF tract from different patterns of a noncomplicated cesarean scar.[4] Helical CT appears to be a valuable tool in depicting a VUF.[5] When a low VUF is present with the communication below the isthmus, CT after IV contrast injection is a good method to show the fistula. When a high VUF is suspected, helical CT with sagittal reformation, performed after hysterography, gives more information about the precise topography of the fistulous tract.[4] Disadvantage of helical CT is administration of IV contrast and additional procedures like hysterography. The MRI as a noninvasive method allowing the avoidance of allergic, nephrotoxic contrast medium and provides a better definition against the surrounding tissues.[6] High resolution T2-weighted MRI clearly demonstrates the hypointense fistula tract and hyperintense endometrial cavity due to stagnation of urine as shown in Figure 1. The MRI can denote the exact position of the fistula and surrounding anatomy [Figure 2]. It is very useful in the diagnosis of Youssef's syndrome with atypical clinical presentation like our case, obviating the need of invasive conventional radiographic contrast examination. The MRI should be suggested as the choice of investigation for VUF, when it is available.
